# Supplementary figures and images for: The impact of biologic agents on cardiovascular risk factors in patients with rheumatoid arthritis: A meta analysis
Source: PLoS One. 2024 Aug 29;19(8):e0306513. doi: 10.1371/journal.pone.0306513 (PMC11361434; doi:10.1371/journal.pone.0306513)

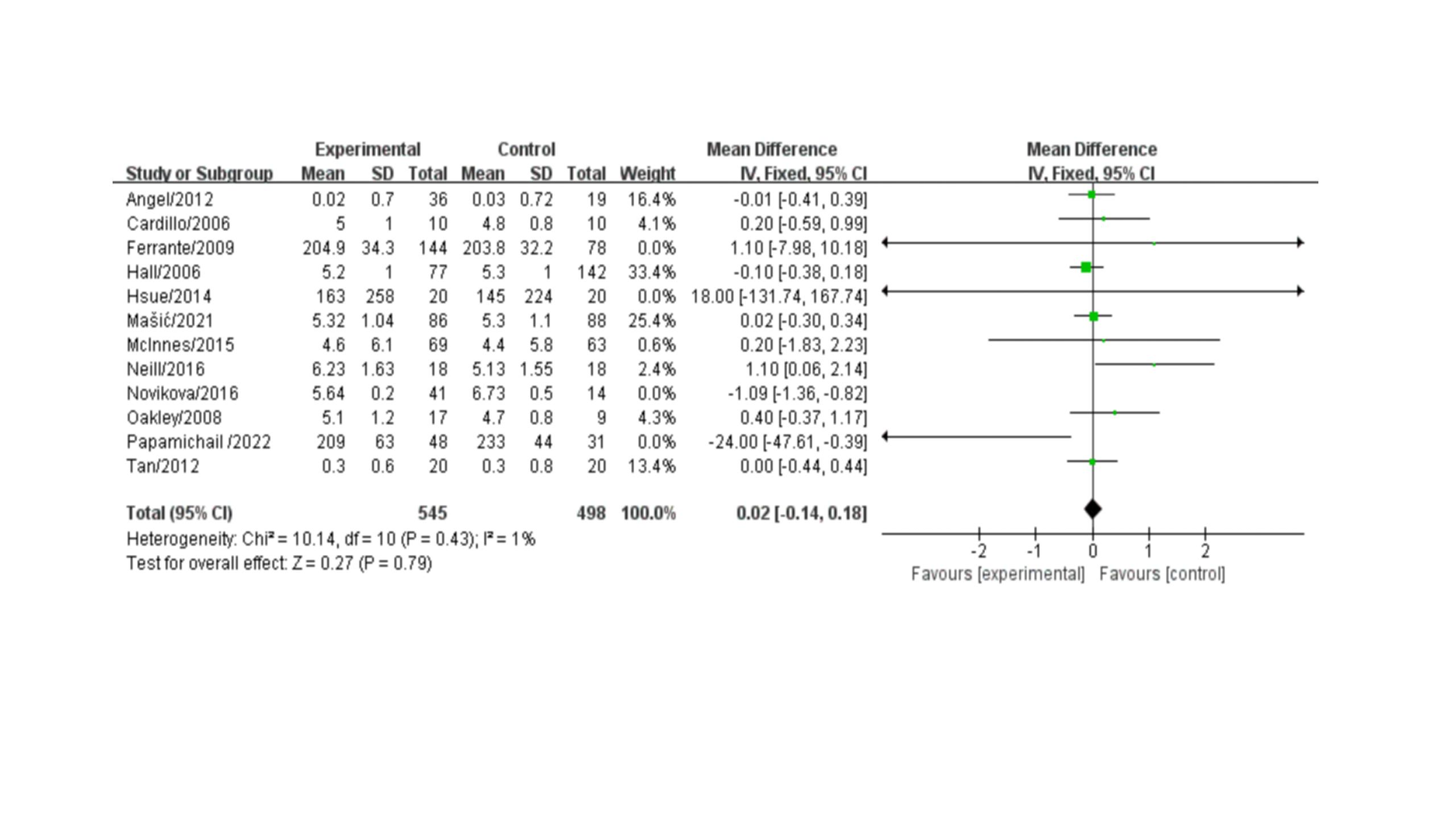

Supplement: S1 Fig — (TIF) [file pone.0306513.s003.tif]

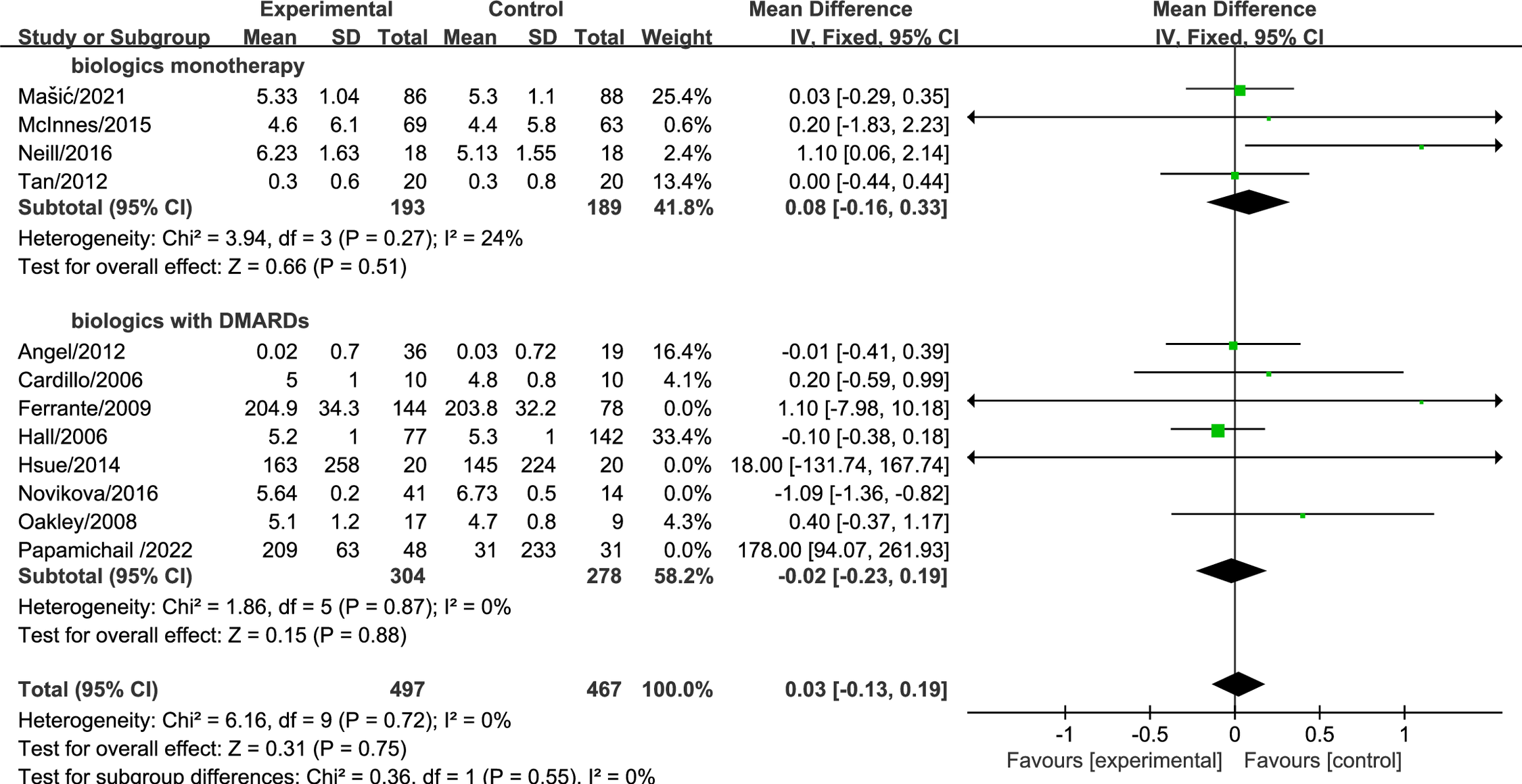

Supplement: S2 Fig — (TIF) [file pone.0306513.s004.tif]

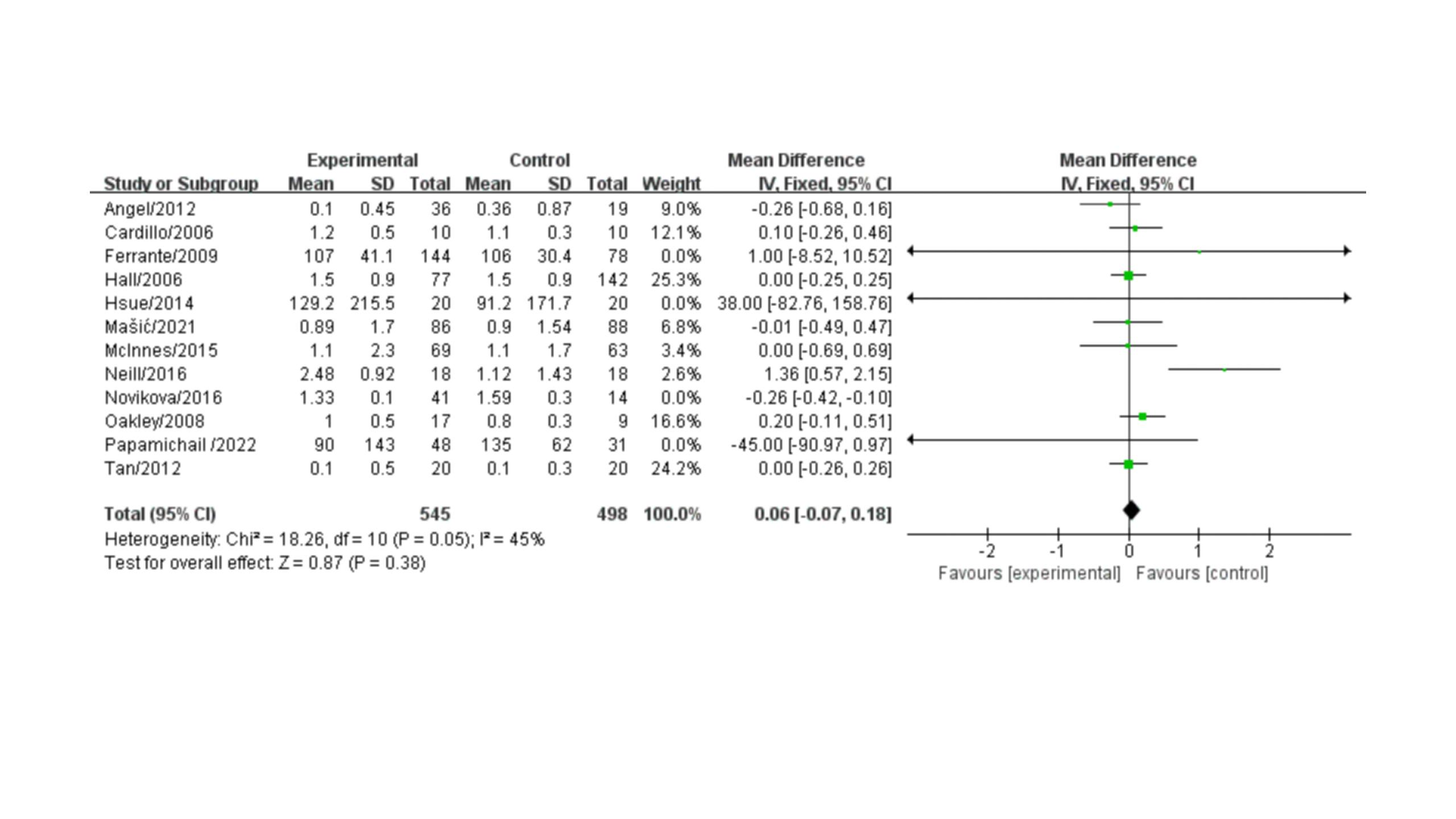

Supplement: S3 Fig — (TIF) [file pone.0306513.s005.tif]

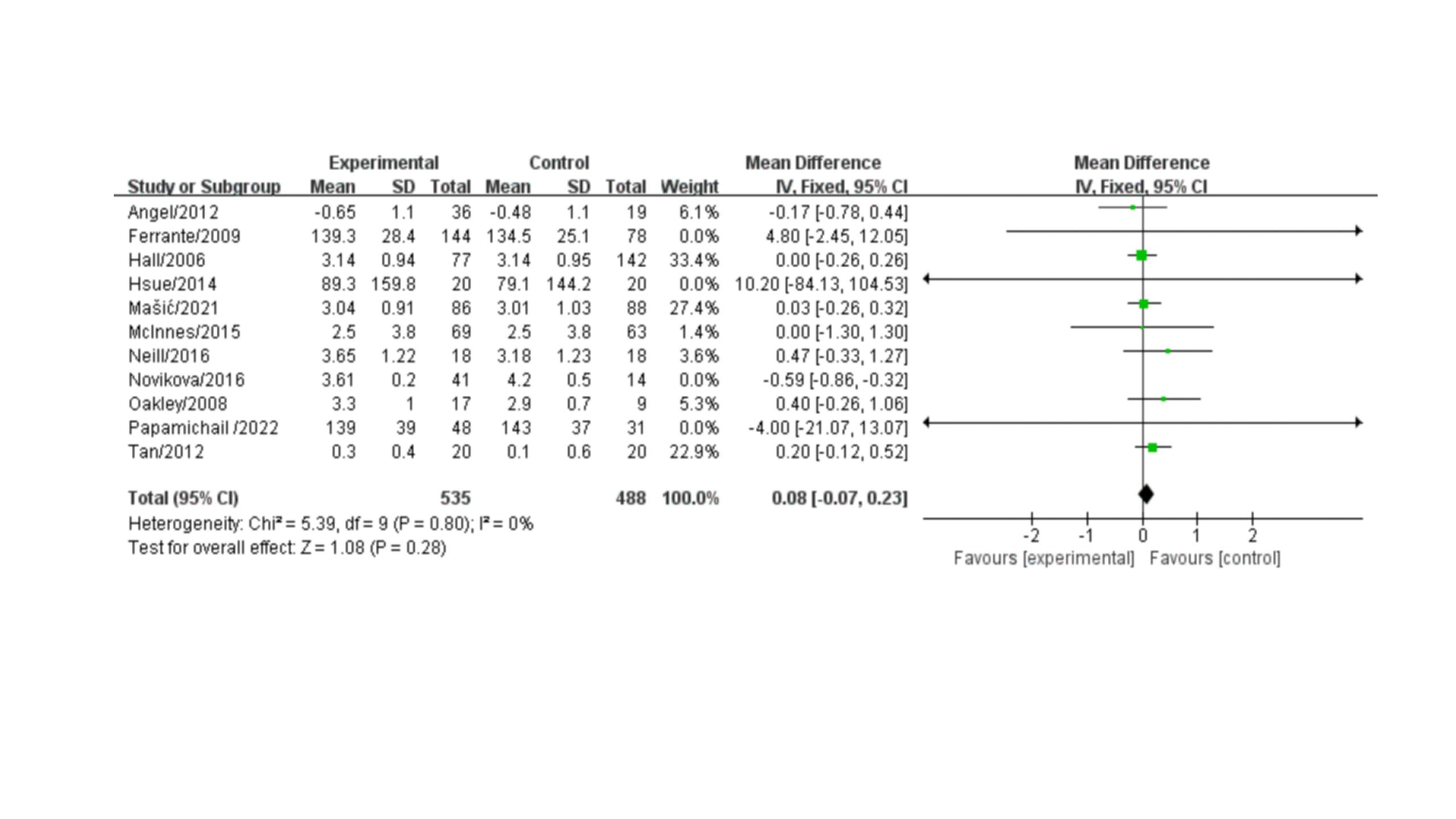

Supplement: S4 Fig — (TIF) [file pone.0306513.s006.tif]

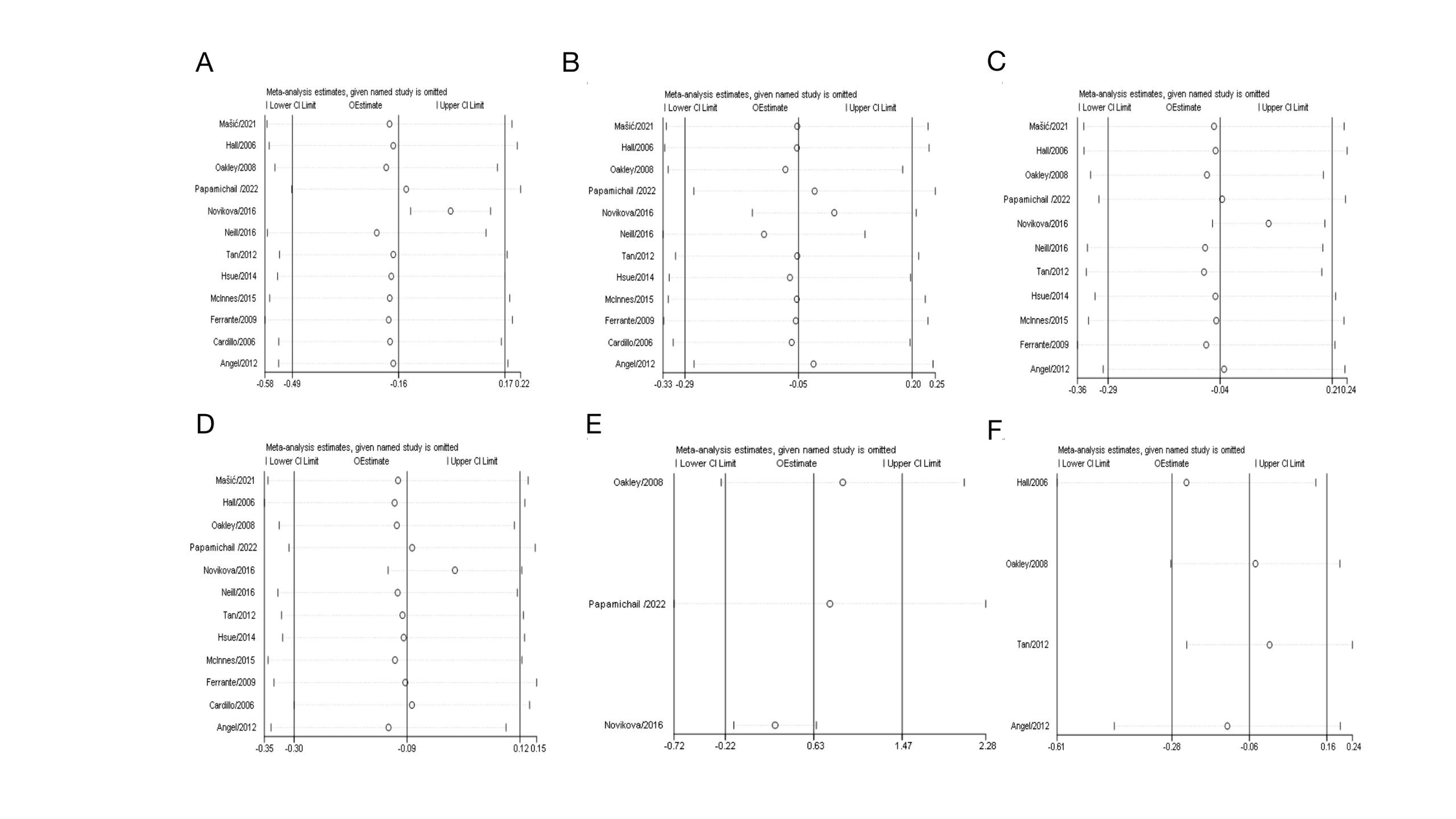

Supplement: S5 Fig — A: Total cholesterol B: triglyceride C: low density lipoprotein D: HDL-C E: medial carotid intima thickness F: PWV. (TIF) [file pone.0306513.s007.tif]

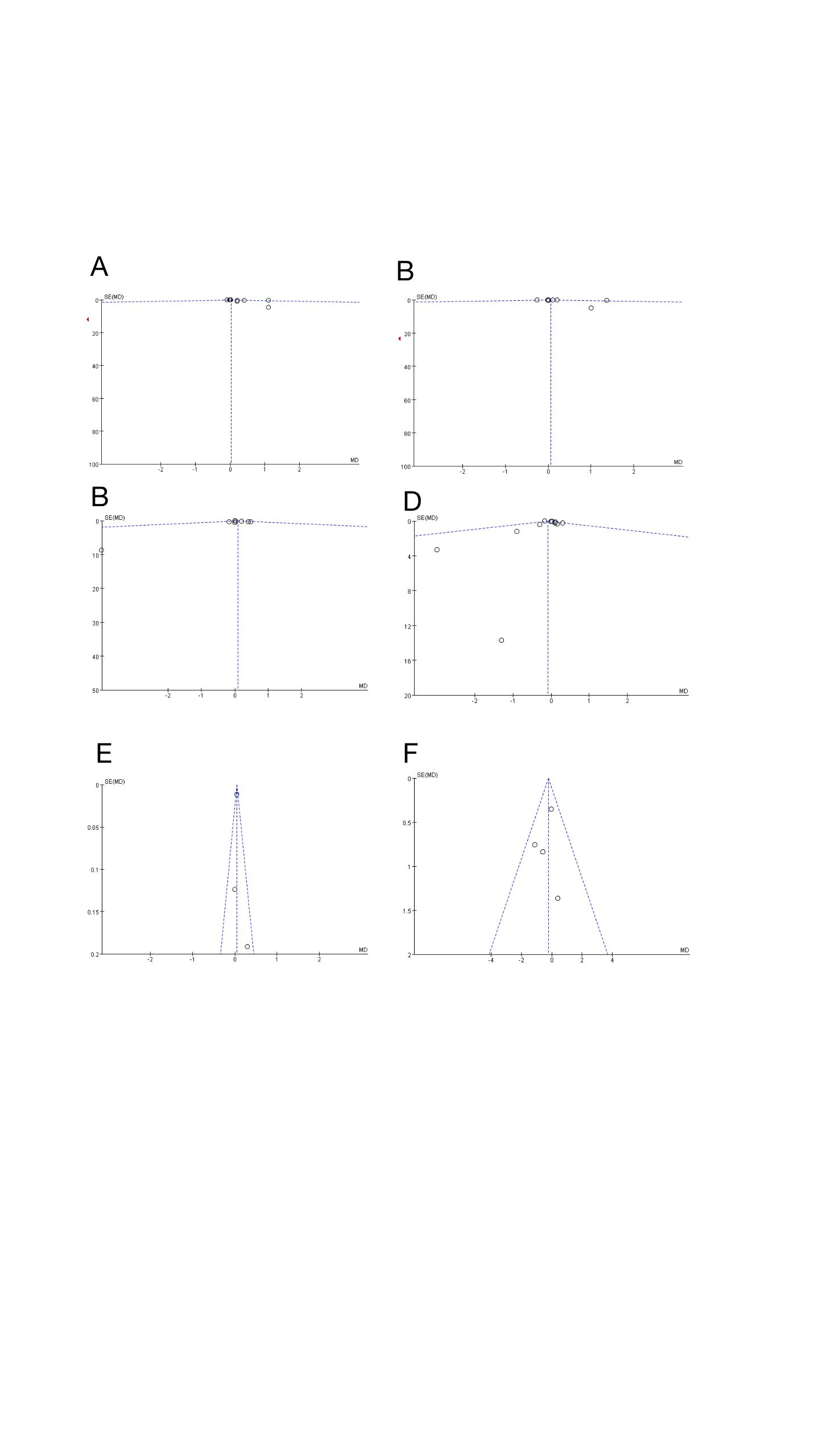

Supplement: S6 Fig — A: Total cholesterol B: triglyceride C: low density lipoprotein D: HDL-C E: medial carotid intima thickness F: PWV. (TIF) [file pone.0306513.s008.tif]
