# Supplementary material for: The impact of biologic agents on cardiovascular risk factors in patients with rheumatoid arthritis: A meta analysis
Source: PLoS One. 2024 Aug 29;19(8):e0306513. doi: 10.1371/journal.pone.0306513 (PMC11361434; doi:10.1371/journal.pone.0306513)
Supplement: Attachment — Submitted filename: Response to Reviewers'.docx [file pone.0306513.s009.docx]

1- **The question of research should be made clear using the PICO model.**

Response to Reviewer: Thank you for your valuable feedback on our paper. We highly appreciate your suggestions and have made the necessary revisions in response to your comments. We have clarified the research question using the PICO model on page 2, lines 25-26, as indicated with red markers. (Objective: This study aims to assess the effects of biologic therapy on cardiovascular risk factors in patients with rheumatoid arthritis (RA) and determine its clinical efficacy). Should there be any other areas that require improvement, please do not hesitate to let us know. We would be more than happy to make further modifications to the manuscript. Thank you once again for your time and professional insights.

**2- The study designs must be mentioned in the inclusion criteria.**

Response to Reviewer: Thank you for your valuable feedback on our paper. We highly appreciate your suggestions and have made the necessary revisions in response to your comments. Based on your recommendation, we have now explicitly mentioned the study designs in the inclusion criteria. We acknowledge that this is an important piece of information that will help readers better understand the scope and methodology of our research. We have mentioned the study designs in the included criteria on page 5, lines 108-109, as indicated with red markers. (All included studies consisted of randomized controlled trials as well as retrospective or prospective observational studies). We sincerely appreciate your guidance and suggestions, and we believe that these modifications will further enhance the accuracy and completeness of our paper. Should there be any other areas that require improvement, please do not hesitate to let us know. We would be more than happy to make further modifications to the manuscript. Thank you once again for your time and professional insights.

**3-Meta analyses and systematic reviews should be registered, and the registration number needs to be used as reference during the submission process.**

Response to Reviewer: Thank you for your valuable feedback on our paper. We highly appreciate your suggestions and have made the necessary revisions in response to your comments. Based on your recommendation, we have registered our meta-analyses and systematic reviews. We have obtained a registration number, which we will now reference during the submission process to ensure transparency and adherence to best practices. We have registered the meta-analyses and systematic reviews, and the registration number has been used as a reference during the submission process. The information can be found on page 3, lines 48-49 (Systematic Review Registration https://www.crd.york.ac.uk/PROSPERO/, CRD42024504911), and page 4, lines 85 (The review proposal was registered with PROSPERO: CRD42024504911.), as indicated with red marker. We sincerely appreciate your guidance and suggestions, and we believe that these modifications will further strengthen the quality and reliability of our paper. Should there be any other areas that require improvement, please do not hesitate to let us know. We would be more than happy to make further modifications to the manuscript. Thank you once again for your time and professional insights.

4- **Causes of heterogeneity aren’t clearly explained.**

Response to Reviewer: Thank you for your valuable feedback on our paper. We highly appreciate your suggestions and have made the necessary revisions in response to your comments. Based on your recommendation, we have provided a clearer explanation of the causes of heterogeneity in our study. We acknowledge that understanding and addressing sources of heterogeneity are crucial for interpreting the results accurately. We have clearly explained the causes of heterogeneity in all results through sensitivity and subgroup analysis, as show on page 11, lines 183-186 ( Following the exclusion of the study conducted by Novikova et al. (2016), the impact of biologic-based treatment on total cholesterol levels was no longer statistically significant (MD = 0.02, 95% CI: [-0.02, 0.18], p = 0.79). Furthermore, the heterogeneity decreased substantially from 81% to 1% (SFigure 1)) and lines 188-201(different treatment approaches; In the subgroup analysis of different drug treatments, no heterogeneity was detected, and the combined effect size suggested a tendency towards a reduction in total cholesterol levels among patients receiving biologics in combination with disease-modifying antirheumatic drugs (DMARDs). However, this observed trend did not reach statistical significance (SFigure 2)), with a red marker. We sincerely appreciate your guidance and suggestions, and we believe that these modifications will further enhance the clarity and robustness of our findings. Should there be any other areas that require improvement, please do not hesitate to let us know. We would be more than happy to make further modifications to the manuscript. Thank you once again for your time and professional insights.

5- **There are some drug names that are not correctly written.**

Response to Reviewer: Thank you for your valuable feedback on our paper. We highly appreciate your suggestions and have made the necessary revisions in response to your comments. Based on your recommendation, we have carefully reviewed the drug names mentioned in our manuscript and rectified any incorrect spellings or errors. We understand the importance of accurately identifying and referencing the drugs used in our study. In our revised version, we have ensured that all drug names are correctly written and have verified their accuracy through appropriate sources, such as official drug databases and reputable references. We sincerely appreciate your guidance and suggestions, and we believe that these modifications will further enhance the accuracy and reliability of our paper. Should there be any other areas that require improvement, please do not hesitate to let us know. We would be more than happy to make further modifications to the manuscript. Thank you once again for your time and professional insights.

6- **Why is leflunomide and mycophenolate mentioned in the search strategy since they are considered conventional synthetic non biologic DMARDs, and if the authors wanted to address additionally conventional synthetic DMARDS where are the other DMARDs including methotrexate and hydroxychloroquine…..etc.**

Response to Reviewer: Thank you for your valuable feedback on our paper. We highly appreciate your suggestions and have made the necessary revisions in response to your comments. Based on your recommendation, we have carefully reviewed the mention of leflunomide and mycophenolate in the search strategy. We acknowledge that these medications are conventional synthetic non-biologic DMARDs and might not align with the scope of our study. In our revised version, we have removed the mention of leflunomide and mycophenolate from the search strategy as they do not directly align with our objectives. We sincerely appreciate your guidance and suggestions, and we believe that these modifications will further enhance the accuracy and relevance of our study. Should there be any other areas that require improvement, please do not hesitate to let us know. We would be more than happy to make further modifications to the manuscript. Thank you once again for your time and professional insights.

7- **The dates for studies included “from …..till” wasn’t mentioned in the search strategy.**

Response to Reviewer: Thank you for your valuable feedback on our paper. We highly appreciate your suggestions and have made the necessary revisions in response to your comments. Based on your recommendation, we have included the specific dates for the study inclusion period in the search strategy. We acknowledge the importance of clearly indicating the time frame during which studies were considered for inclusion. We have added the dates for studies in the search strategy on page 5, lines 90 ,as indicated with red marker (The search was conducted until May 2023). We sincerely appreciate your guidance and suggestions, and we believe that these modifications will further enhance the clarity and comprehensiveness of our study. Should there be any other areas that require improvement, please do not hesitate to let us know. We would be more than happy to make further modifications to the manuscript. Thank you once again for your time and professional insights.

**8- Combining studies with different treatment strategies like studies using biologics monotherapy versus biologics with conventional DMARDs makes the meta-analysis will yield heterogenous data.**

Response to Reviewer: Thank you for your valuable feedback on our paper. We highly appreciate your suggestions and have carefully considered the issue raised. You are correct in pointing out that combining studies with different treatment strategies, such as biologics monotherapy versus biologics with conventional DMARDs, may introduce heterogeneity into the meta-analysis. We acknowledge the potential impact of this heterogeneity on the pooled data. In our revised version, we have taken your suggestion into account and conducted a subgroup analysis to address the heterogeneity arising from different treatment strategies. This approach allows us to assess the effects of these distinct treatment approaches separately, providing a more comprehensive understanding of the results. We have added the subgroup analysis comparing biologics monotherapy versus biologics with conventional DMARDs in the results section on page 11, lines 197-201 (In the subgroup analysis of different drug treatments, no heterogeneity was detected, and the combined effect size suggested a tendency towards a reduction in total cholesterol levels among patients receiving biologics in combination with disease-modifying antirheumatic drugs (DMARDs). However, this observed trend did not reach statistical significance (SFigure 2)), page 12, lines 220-222 (When stratified by different treatment approaches, biologic treatment of RA was found to effectively reduce triglyceride levels when using biologics monotherapy (MD = -0.16, 95% CI [-0.28, -0.04], P = 0.01) (Figure 6)), and page 13, lines 232-233(The subgroup analyses based on different treatment methods and treatment duration did not reveal statistically significant findings (Figure 7-8)), as indicated with red markers. We sincerely appreciate your guidance and suggestions, and we believe that the inclusion of subgroup analysis will improve the robustness and accuracy of our meta-analysis. Should there be any other areas that require improvement, please do not hesitate to let us know. We would be more than happy to make further modifications to the manuscript. Thank you once again for your time and professional insights.
