# Supplementary material for: The impact of biologic agents on cardiovascular risk factors in patients with rheumatoid arthritis: A meta analysis
Source: PLoS One. 2024 Aug 29;19(8):e0306513. doi: 10.1371/journal.pone.0306513 (PMC11361434; doi:10.1371/journal.pone.0306513)
Supplement: Attachment — Submitted filename: Response to Reviewers.docx [file pone.0306513.s010.docx]

**1-Comprehensive Assessment of Cardiovascular Risk: it is crucial to consider a wide range of cardiovascular risk factors beyond lipid levels, such as the use of aspirin, statins, antihypertensive drugs, smoking status, presence of diabetes,familiarity and obesity. These factors play a significant role in determining overall cardiovascular risk in patients with rheumatoid arthritis (RA).**

Response to Reviewer: Thank you for your valuable feedback on our paper. We highly appreciate your suggestions and have made the necessary revisions in response to your comments. We have revised the limitations section in the manuscript and expanded our discussion on broader cardiovascular risk factors that should be considered in addition to lipid levels. Specifically, we have addressed the importance of assessing cardiovascular risk factors such as the use of aspirin, statins, antihypertensive drugs, smoking status, presence of diabetes, familiarity, and obesity in patients with rheumatoid arthritis (RA). We emphasize that these factors play a significant role in determining the overall cardiovascular risk in this patient population. By incorporating these factors into our analysis, we can gain a more comprehensive understanding of the relationship between rheumatoid arthritis and cardiovascular disease. In the newly added limitation section, we have highlighted the need for future studies to comprehensively assess these factors in order to provide a more accurate assessment of cardiovascular risk in patients with RA. This will enhance our understanding of the complex interplay between rheumatoid arthritis and cardiovascular disease and help inform clinical decision-making.

Here is an updated version:

“One of the primary limitations of our study is that although we focus on lipid levels as cardiovascular risk factors in RA, it is important to note that there are other significant risk factors that should be taken into account. Factors such as the use of aspirin, statins, antihypertensive drugs, smoking status, presence of diabetes, familiarity, and obesity play a substantial role in determining the overall cardiovascular risk in these patients. Future studies should aim to comprehensively assess these factors to provide a more accurate evaluation of cardiovascular risk in RA.”

You may also refer to the manuscript (page 16, line 309-315) for more details, which have been highlighted in red.

Once again, we thank you for your insightful comments, which have greatly improved the quality of our paper. We remain committed to further enhancing our research and addressing any additional concerns you may have.

Thank you for considering our response.

**2- Disease Severity and Cardiovascular Risk: the differences in intima-media thickness (IMT) between patients on biologic therapy and control groups could be influenced by the baseline disease severity and overall cardiovascular risk profile of the patients. Patients not on biologic therapy may have a milder disease activity, leading to potentially lower cardiovascular risk compared to those on biologic agents. This confounding factor should be carefully considered and addressed in the analysis.**

Response to Reviewer: Thank you for your valuable feedback on our paper. We highly appreciate your suggestions and have made the necessary revisions in response to your comments. We have revised the limitations section in the manuscript to address the concern you raised regarding the potential influence of disease severity and overall cardiovascular risk profile on the differences in intima-media thickness (IMT) observed between patients on biologic therapy and the control groups. We acknowledge that patients not on biologic therapy may have a milder disease activity, which could potentially result in a lower cardiovascular risk compared to those on biologic agents. This confounding factor is indeed an important consideration and needs to be carefully addressed in our analysis. In the limitation section, we emphasize the need for future studies to account for and analyze the baseline disease severity and overall cardiovascular risk profiles of patients. By doing so, we can provide a more comprehensive understanding of the relationship between biologic therapy, disease severity, and cardiovascular risk. This will help mitigate the potential confounding effects and enhance the accuracy of our findings.

Here is an updated version:

“Furthermore, the observed differences in intima-media thickness (IMT) between patients on biologic therapy and control groups may be influenced by the baseline disease severity and overall cardiovascular risk profile of the patients. Patients not receiving biologic therapy might have milder disease activity, potentially leading to a lower cardiovascular risk compared to those on biologic agents. This confounding factor should be carefully considered and addressed in future analyses to ensure an accurate assessment of the impact of biologic therapy on cardiovascular risk.”

You may also refer to the manuscript (page 16-17, line 315-322) for more details, which have been highlighted in red.

Once again, we thank you for your insightful comments, which have greatly improved the quality of our paper. We remain committed to further enhancing our research and addressing any additional concerns you may have.

Thank you for considering our response.

**3-Inflammatory Influence: chronic inflammation in RA can lead to alterations in lipid metabolism, including changes in lipid levels that may not align with the expected cardiovascular risk profile. Biologic therapy targeting inflammation may improve lipid profiles but may not always translate to a reduced cardiovascular risk due to the complex interplay between inflammation and lipid metabolism (lipid paradox).**

Response to Reviewer: Thank you for your valuable feedback on our paper. We highly appreciate your suggestions and have made the necessary revisions in response to your comments.We have revised the limitations section in the manuscript to address the concern you raised regarding the inflammatory influence in rheumatoid arthritis and its impact on lipid metabolism. We acknowledge that chronic inflammation in RA can lead to alterations in lipid levels, which may not align with the expected cardiovascular risk profile. While biologic therapy targeting inflammation can potentially improve lipid profiles, it may not always translate to a reduced cardiovascular risk due to the complex interplay between inflammation and lipid metabolism, commonly known as the "lipid paradox." In the limitation section, we discuss the need to consider the influence of chronic inflammation on lipid metabolism and its implications for assessing cardiovascular risk in RA patients. We emphasize that although biologic therapy may positively impact lipid profiles, the overall cardiovascular risk reduction may be affected by factors related to the underlying inflammatory processes. We highlight the importance of further research to better understand this intricate relationship between inflammation, lipid metabolism, and cardiovascular risk in RA.

Here is an updated version:

“ It is also worth noting that chronic inflammation associated with RA can lead to alterations in lipid metabolism, resulting in changes in lipid levels that may not align with the expected cardiovascular risk profile. While biologic therapy targeting inflammation may improve lipid profiles, the complex interplay between inflammation and lipid metabolism, known as the "lipid paradox," may limit the translation of improved lipid profiles into reduced cardiovascular risk. Future studies should further investigate this interrelationship to better understand how inflammation affects lipid metabolism and its implications for assessing cardiovascular risk in RA.”

You may also refer to the manuscript (page 17, line 322-329) for more details, which have been highlighted in red.

Once again, we thank you for your insightful comments, which have greatly improved the quality of our paper. We remain committed to further enhancing our research and addressing any additional concerns you may have.

Thank you for considering our response.

**4-Long-Term Observations: Evaluating the impact of biologic therapy on cardiovascular risk factors over a longer period is essential to capture any potential changes or trends that may not be evident in a short-term study. Longitudinal studies can provide a more comprehensive understanding of the effects of biologic agents on cardiovascular health in RA patients. The lipid paradox highlights the importance of considering long-term cardiovascular risk assessment beyond lipid levels alone. While improvements in lipid profiles with biologic therapy are beneficial, the overall impact on cardiovascular outcomes may be influenced by a combination of traditional and non-traditional risk factors.**

Response to Reviewer: Thank you for your valuable feedback on our paper. We highly appreciate your suggestions and have made the necessary revisions in response to your comments. We have revised the limitations section in the manuscript to address the importance of long-term observations in evaluating the impact of biologic therapy on cardiovascular risk factors in patients with rheumatoid arthritis. We acknowledge that assessing these effects over a longer period is crucial to capture any potential changes or trends that may not be evident in short-term studies. Longitudinal studies can provide a more comprehensive understanding of the effects of biologic agents on cardiovascular health in RA patients. Furthermore, we discuss the significance of considering long-term cardiovascular risk assessment beyond lipid levels alone, as highlighted by you. We recognize the lipid paradox, which emphasizes the need to evaluate the overall impact of biologic therapy on cardiovascular outcomes considering a combination of both traditional and non-traditional risk factors. While improvements in lipid profiles resulting from biologic therapy are beneficial, other factors may influence the overall cardiovascular outcomes in RA patients. In the limitation section, we emphasize the necessity of conducting longitudinal studies to assess the long-term impact of biologic therapy on cardiovascular risk factors in RA. By considering a broader range of risk factors and incorporating data from extended observation periods, we can gain a more comprehensive understanding of the complex relationship between biologic therapy, cardiovascular risk factors, and outcomes in RA patients.

Here is an updated version:

“Lastly, the short-term nature of our study limits our ability to capture any potential long-term changes or trends related to the effects of biologic therapy on cardiovascular risk factors in RA patients. Longitudinal studies are essential for obtaining a more comprehensive understanding of the long-term impact of biologic agents on cardiovascular health in this population. Additionally, incorporating a broader range of traditional and non-traditional risk factors is crucial for a more holistic evaluation of cardiovascular outcomes in RA patients.”

You may also refer to the manuscript (page 17, line 329-335) for more details, which have been highlighted in red.

Once again, we thank you for your insightful comments, which have greatly improved the quality of our paper. We remain committed to further enhancing our research and addressing any additional concerns you may have.

Thank you for considering our response.
